# Trisomy 13, home health-care and multidisciplinary approach: Case report

**DOI:** 10.1590/1984-0462/2025/43/2024018

**Published:** 2024-10-04

**Authors:** Patrícia Zschaber Anacleto, Virginia Araújo de Sousa, Julliane V Joviano-Santos

**Affiliations:** a Faculdade Ciências Médicas de Minas Gerais, Post-Graduate Program in Health Sciences, Belo Horizonte, MG, Brazil.; b Instituto de Previdência dos Servidores do Estado de Minas Gerais, Belo Horizonte, MG, Brazil.

**Keywords:** Trisomy 13 syndrome, Patau’s syndrome, Home nursing, Perinatal care, Síndrome da trissomia do cromossomo 13, Síndrome de Patau, Assistência domiciliar, Assistência perinatal

## Abstract

**Objective:**

To recognize and address Patau’s syndrome, despite its rarity and associated low life expectancy, through the presentation of a case study of a 2-year-old patient receiving Home Care services.

**Case description:**

We present a female patient who defied the odds with a prolonged survival, possible due to Home Care. She was delivered via cesarean section at 31 weeks + 4 days due to restricted uterine growth. The mother, aged 36, had received proper prenatal care and was in good health. The diagnosis of Patau’s syndrome was confirmed through karyotyping after birth. Despite the severe clinical nature of the case, the patient, now with two years old, receives specialized home-based care, supported by a tracheostomy and gastrostomy. A dedicated 24-hour nursing technician ensures continuous monitoring, and the patient benefits from regular medical check-ups, physiotherapy five times a week, weekly speech therapy sessions, monthly consultations with a nutritionist, and ongoing psychological support for her family members.

**Comments:**

This multidisciplinary approach has resulted in a slight motor response, highlighting the positive impact of comprehensive care on her overall well-being. The existence of a robust support network for families facing similar challenges is crucial, and a multidisciplinary care can effectively prevent complications associated with this impactful syndrome.

## RESUMO

**Objetivo:**

Reconhecer e abordar a síndrome de Patau, apesar de sua raridade e da baixa expectativa de vida associada, por meio da apresentação de um estudo de caso de uma paciente de dois anos que recebe cuidados em casa por intermédio de serviços de *home care*.

**Descrição do caso:**

Apresentamos uma paciente do sexo feminino que desafiou as probabilidades, com sobrevivência prolongada devida, possivelmente, ao *home care*. Ela nasceu por cesariana, com 31 semanas + 4 dias, em razão da restrição do crescimento uterino. A mãe, de 36 anos, recebeu cuidados pré-natais adequados e gozava de boa saúde. O diagnóstico da síndrome de Patau foi confirmado por meio de cariótipo após o nascimento. Apesar da gravidade clínica do caso, a paciente, hoje com dois anos, recebe atendimento domiciliar especializado, apoiado por traqueostomia e gastrostomia. Um técnico de enfermagem dedicado 24 horas garante acompanhamento contínuo, e a paciente beneficia-se de *check-ups* médicos regulares, fisioterapia cinco vezes por semana, sessões semanais de fonoaudiologia, consultas mensais com nutricionista e apoio psicológico contínuo aos familiares.

**Comentários:**

Esta abordagem multidisciplinar resultou em melhora motora discreta, destacando o impacto positivo do cuidado integral no seu bem-estar geral. A existência de uma rede robusta de apoio às famílias que enfrentam desafios semelhantes é crucial, e um cuidado multidisciplinar pode prevenir eficazmente as complicações associadas a esta impactante síndrome.

## INTRODUCTION

Trisomy 13, also known as Patau’s syndrome, is an autosomal genetic disease. It ranks as the third most common chromosomal aneuploidy at birth, following trisomies 18 and 21, known as Edwards’ syndrome and Down’s syndrome, respectively.^1,2,3^ The genetic description of Patau’s syndrome dates back to 1960, when it was studied by Patau et al.^4^ This condition arises due to a non-disjunction of germ cells during meiosis I or II, leading to the presence of three chromosomes 13. Trisomy 13 can manifest in complete, partial, or mosaic forms, with full expression being the most common clinically observed form. It is worth noting that maternal age greater than 35 years is considered a significant risk factor for the occurrence of this syndrome.^5^

Patau’s syndrome leads to a range of malformations, including those affecting the central nervous system, heart, kidneys, facial features, limbs, and gastrointestinal tract, as well as auditory and ocular anomalies.^6,7^ During prenatal care, chromosomal abnormalities associated with Patau’s syndrome can be detected through ultrasounds, a non-invasive screening method that poses minimal risk to the fetus. However, a final diagnosis is confirmed through a karyotype analysis.^8^

Trisomy 13 occurs in approximately 1 out of 10,000 to 20,000 live births, and is associated with a high prenatal mortality rate, with around 95% of affected pregnancies not reaching full term. Due to its low occurrence, it is classified as a rare condition.^3,7,9^ Despite its rarity and the associated low life expectancy, it is essential to recognize and address Patau’s syndrome. In this context, we present a case study of a 2-year-old patient who receives care at home through Home Care services. Our report emphasizes the significance of daily home-based care that could be contributing to the patient’s survival provided by a multidisciplinary team and innovative techniques.

## CASE REPORT

This study was approved by the Ethics in Research Committee (CAAE# 68944723.0.0000.5134). The patient under consideration is a 2-year-old girl, born prematurely at 31 weeks + 4 days of gestation via cesarean section due to intrauterine growth restriction in Belo Horizonte, Brazil. At birth, she weighed 1095 kg and received Apgar scores of 8 and 9. Notably, her parents are unrelated, healthy, and also have a healthy 4-year-old daughter. There are no reported comorbidities in the family history. Throughout her pregnancy, the mother had six prenatal consultations, during which no maternal serological changes were observed. The mother did not take any medications during pregnancy and was not exposed to any teratogens. Additionally, in the week preceding the birth, the mother underwent a course of corticosteroids. An overview of characteristics observed in individuals exhibiting Patau’s syndrome in contrast to the traits present in our patient was displayed in Table 1; adapted from.^10^

**Table 1:** Overview of characteristics observed in individuals exhibiting Patau’s syndrome in contrast to the traits present in our patient^11^.

Physical findings	% of cases	Our patient
Broad nasal bridge and Short nose	100.0	+
Sloping forehead and Telangiectatic nevus	100.0	-
Upturned nares	100.0	+
Uterine anomalies	100.0	-
Depressed nasal bridge	87.5	+
Cryptorchidism	85.7	-
Upslanting palpebral fissures	85.7	+
Low-set ears	85.0	+
Epicanthal folds and Microtia	83.3	+
Skin redundancy	83.3	-
Apnea and Frequent respiratory infections	80.0	+
Finger clinodactyly	80.0	+
Low posterior hairline and Pigmentary abnormalities	80.0	-
Short neck	80.0	+
Small forehead	80.0	-
Tapered fingers	80.0	+
Patent ductus arteriosus	77.8	+
Cleft palate	77.3	+
Facial asymmetry and Hearing loss	75.0	-
Flat occiput and High forehead	75.0	+
Poor suck/swallow	75.0	-
Malformed ears	75.0	+
Seizures	73.7	+
Hypotelorism and Joint contractures	71.4	+
Vertebral anomalies	71.4	-
Widely spaced nipples	71.4	+
Ventricular septal defect	70.0	+
Micrognathia	69.2	+
Clenched hands	66.6	-
Finger camptodactyly	63.6	-
High palate	63.6	+
Umbilical hernia	62.5	-
Cleft lip	61.5	+
Downslanting palpebral fissures and Hypoplastic nose	60.0	+
Pterygium	60.0	-
Two-vessel umbilical cord	60.0	+
Atrial septal defect	55.6	-
Rib anomalies	51.1	-
Finger brachydactyly and Thin upper lip	50.0	-
Cognitive/developmental findings		
Speech delay	100.0	+
Intellectual disability	80.9	+
Global developmental delay	71.4	+

During prenatal care, an obstetric ultrasound at 16 weeks of gestational age revealed a cleft lip, and a morphological ultrasound at 25 weeks identified a cleft palate. Patau’s syndrome was confirmed only after the patient’s birth through karyotype analysis, which showed 47,XX,+13. Upon birth, the patient experienced early respiratory distress in the delivery room, necessitating immediate transfer to the Neonatal Intensive Care Unit (ICU). She was placed on continuous positive airway pressure (CPAP) using a Baby Puff device. Within four hours of birth, the patient received a dose of surfactant. She remained on mechanical ventilation for 5 days before being extubated and placed in a baby incubator. Due to the patient’s underlying condition, attempts to adapt CPAP or Non-invasive ventilation (NIV) were not successful.

At 7 days of life, the newborn had to be reintubated. Despite two attempts to discontinue mechanical ventilation, the patient was not able to tolerate it, experiencing frequent episodes of cyanosis, decreased chest expansion, and difficulty breathing. As a result, a tracheostomy was performed using a 4.0 cannula without a cuff. Unfortunately, the patient remained unable to be weaned from mechanical ventilation and continued to exhibit episodes of chest locking, oxygen saturation drops, and showed radiological signs of pulmonary dysplasia. Additionally, the patient underwent gastrostomy and received parenteral nutrition for a period of 6 days.

During the hospitalization, the newborn underwent several diagnostic tests. An amplified neonatal screening test was performed, revealing no abnormalities. Additionally, an ophthalmological evaluation included a bilateral red reflex test, which indicated a slight decrease in the macular reflex along with thinned vessels in both eyes. Imaging tests conducted during the hospital stay included an abdominal ultrasound, which detected a nodular liver lesion (interpreted as a hematoma) and minimal bilateral pelvic ectasia. An echocardiogram showed the presence of an ostium secundum-type atrial septal defect, mild tricuspid regurgitation, normal heart chamber sizes, and preserved biventricular systolic function. The pulmonary arterial systolic pressure was 28 mmHg.

The patient received packed red blood cells to address multifactorial anemia (hemoglobin: 6.3). Medical procedures performed included a tracheostomy replacement with a 4.0 cannula equipped with a balloon and a gastrostomy exchange for a Mic-key 15 FR 0.8. Following the hospitalization period, the infant was discharged for continued care under the Home Care program.

Despite the severity of the case, the patient, who is now two years old, receives dedicated care at home through the Home Care program, which could be contributing to patient survival. She relies on a tracheostomy and gastrostomy for her medical needs, and has the support of a 24-hour nursing technician. Additionally, she undergoes comprehensive medical care, including weekly medical check-ups, physiotherapy sessions five times a week, speech therapy once a week, monthly consultations with a nutritionist, and psychological support for her family members.

Thanks to this multidisciplinary approach, the patient has shown slight improvements in her motor responses. Her convulsive seizures are managed with medication, and her last hospitalization occurred in January 2023, when she was admitted due to pneumonia. Despite the challenges, the patient continues to receive consistent and compassionate care, allowing her to stay at home with her family.

## DISCUSSION

It is believed that the increased survival rate in the case described is fully correlated to the Home Care offered to patients in the form of home hospitalization, in addition to invasive treatments such as tracheostomy and gastrostomy administered early in the patient’s life. The profound impact of this approach on patient outcomes cannot be overlooked. In the field of pediatric medicine, there has been a growing recognition of the pivotal role played by home-based care and multidisciplinary teams in enhancing the survival rates and quality of life for medically complex patients, including patients with Patau’s syndrome.^11,12,13,14^

The increased survival rate observed in the aforementioned case can be possibly attributed to the holistic care provided within the patient’s home environment. Home hospitalization, as a form of specialized medical care delivered in the comfort of the patient’s residence, not only ensures continuous monitoring but also facilitates emotional and psychological support from the patient’s immediate family, thereby creating a conducive healing environment.^15^ Moreover, the use of invasive treatments, i.e., tracheostomy and gastrostomy, can be associated with improved respiratory and nutritional support, leading to enhanced overall well-being and longevity. Within the context of the growing number of reported cases of patients with Patau’s syndrome experiencing long-term survival, it becomes imperative to acknowledge and address this phenomenon in medical discourse. By elucidating this, researchers can underscore its clinical significance and extend its relevance beyond specialized domains, like Home Care. Highlighting this trend in new articles would not only emphasize its significance but also broaden its applicability. For instance, Table 2 presents potential complications, adaptations, and necessary care for patients experiencing prolonged survival, offering a structured overview, adapted from.^16,17^

**Table 2:** Complications, adaptations, and care requirements for prolonged patient survival^16,17^.

Domains	Challenges
Complications/Changes	Renal issues
	Cardiac malformations
	Brain malformations
	Respiratory problems
	Small Intestine obstructions
Necessary Care	Patient communication
	Surgical interventions
	Physiotherapy
	Palliative care
	Tracheostomy
	Gastrostomy

Furthermore, the success of such complex medical cases is intricately linked to the collaboration of a multidisciplinary healthcare team. A multidisciplinary approach involves the active participation of healthcare professionals from various disciplines, including physicians, nurses, therapists, social workers, and nutritionists, working collaboratively to address the diverse needs of the patient.^18^ This interdisciplinary synergy ensures comprehensive and personalized care, tailored to the unique requirements of the patient, thereby maximizing the effectiveness of the treatment regimen.

For example, the constant presence of a nursing technician, available 24 hours a day, played a pivotal role in alleviating the burden on the family and ensuring the patient’s well-being. This continuous monitoring, facilitated through devices such as an oximeter, provided essential medical oversight while offering invaluable support to the patient. By relieving families of the responsibility of continuous care, nursing technicians significantly contributed to the emotional and physical relief of caregivers.

It is important to highlight that a structured healthcare protocol was implemented, which involved regular weekly visits by a qualified medical doctor. These scheduled visits served a dual purpose: first, they allowed for timely medical intervention in cases of complications, thereby mitigating the need for unnecessary hospitalizations. Second, the doctor’s presence at home enabled the early initiation of essential treatments, such as antibiotic therapy, tailored to the patient’s specific condition.

This proactive approach ensured prompt medical attention and contributed to the overall management of the patient’s health, preventing the worsening of the condition. Besides, the comprehensive care provided at home extended beyond immediate medical interventions. The healthcare team conducted necessary examinations and monitored the patient’s other underlying pathologies, addressing multiple health concerns within the familiar and supportive environment of the patient’s home. This approach enhanced the overall quality of care by encompassing a holistic understanding of the patient’s health status. Equally significant was the sense of security instilled in the patient’s family through the availability of consistent communication channels. The opportunity for families to seek clarification of doubts and receive frequent guidance from healthcare professionals fostered a supportive and trusting relationship. This ongoing communication not only enhanced the family’s confidence in the care being provided but also ensured their active participation in the patient’s well-being, thereby creating a collaborative and patient-centered care environment.

A Pediatric neurologist helped to control the seizures associated with the use of the anticonvulsant. The implementation of this type of intervention can effectively control different patient conditions, as already described.^19^ Similarly, a thoracic surgeon performed frequent cannula changes every three months, preventing tracheitis and ensuring the patient’s respiratory health. The gastrostomy underwent scheduled changes every six months at most, being a proactive measure preventing infections. Cutting-edge medical equipment, such as the Trilogy evo mechanical ventilator, was utilized to maintain the patient’s daily breathing requirements within the home setting. This advanced technology not only facilitated mechanical ventilation but also significantly contributed to the patient’s overall respiratory stability and quality of life.^20,21,22^ Daily respiratory and motor physiotherapy were administered to maintain stable respiratory conditions associated with medications. Specialized maneuvers were performed to prevent an increase and impaction of secretions in the lungs, ensuring optimal respiratory function. Motor physiotherapy interventions were implemented to prevent the patient from developing stiffness and atrophy due to prolonged immobilization, stimulating body movement and enhancing overall mobility. The active involvement of subspecialist doctors, coupled with the use of state-of-the-art medical equipment and the dedicated efforts of a multidisciplinary team, resulted in a comprehensive and individualized home-based care model.

The nursing team provided essential guidance to prevent skin injuries and bedsores while maintaining a vigilant focus on the patient’s overall care. They offered valuable advice on caregiver hygiene to minimize the risk of infectious processes. Additionally, the team ensured the patient received proper nutrition through age-specific formulas. To maintain an ideal weight and address specific nutritional deficiencies, supplements such as ferrous sulfate and vitamin D were administered. It was well understood that addressing issues related to overweight and obesity was crucial, as these conditions could exacerbate existing health problems.^23,24^ Besides, the speech therapist played a crucial role in assisting the patient with managing their saliva, thereby preventing sialorrhea and minimizing the risk of micro aspirations. Occupational therapy focused on enhancing the patient’s sensory skills improved the overall development. The psychology team provided invaluable emotional support to the family, acknowledging the challenges of their atypical life and helping them cope, thereby alleviating potential emotional stress. Recognizing the significance of support networks, family members and friends were instrumental in providing comfort and solace to the parents during this difficult period.

An established routine was in place, involving weekly medical care, physiotherapy five times a week, speech therapy three times a week, and occupational therapy once a week. This structured approach had a positive impact on improving survival rates and increasing life expectancy. Patient care by a multidisciplinary team in the home environment and with family support can possibly increase patient survival. Through these interventions, the evidence was challenged, and, most importantly, the team managed to provide a good quality of life for the patient, despite all limitations. In conclusion, this comprehensive care strategy successfully avoided unnecessary hospitalizations, minimized painful procedures such as punctures, and ensured the patient’s clinical stability. There has been a notable increase in reported cases of individuals with Patau’s syndrome living longer. This challenges previous assumptions about the condition’s severity and lifespan. Improved medical care, early detection, and supportive interventions have contributed to this trend. These cases of longer survival not only offer hope to affected families but also present unique opportunities for researchers and healthcare professionals to explore novel approaches to care and support tailored to the evolving needs of these individuals.
